# An integrated framework of ground source heat pump utilisation for high-performance buildings

**DOI:** 10.1038/s41598-023-27704-2

**Published:** 2023-01-07

**Authors:** Hong Xian Li, Daniel E. Okolo, Amir Tabadkani, Tony Arnel, Dongming Zheng, Long Shi

**Affiliations:** 1 School of Architecture and Built Environment, Deakin University, Geelong, Australia; 2 Stantec, Level 3/52 Merivale St., Brisbane, QLD Australia; 3 Faculty of Science Engineering and Built Environment, Deakin University, Geelong, Australia; 4 Department of Civil and Environmental Engineering, University of Alberta, Edmonton, Canada; 5 School of Engineering, RMIT University, Melbourne, Australia

**Keywords:** Civil engineering, Energy infrastructure, Energy and society

## Abstract

CO_2_ emissions from building operations have increased to their highest level globally, moving away from the Paris Agreement goal of below 2 °C. While geothermal is recognised as a promising renewable source, the lack of an integrated framework guiding investigating ground source heat pumps for building operations, along with the incapability of well-known simulation tools in accurately capturing ground thermal performance, hinders its application. This research aims to unlock ground source heat pumps for building operations through an integrated framework, including an overarching improved U.S. National Renewable Energy Laboratory (NREL) monitoring guideline, a sensor-based monitoring prototype, and a g-function-based simulation approach. This research proposes amendments and improvements to the NREL guideline for monitoring geothermal energy by separating *Thermal Energy Net Production* from *Thermal Energy Gross Production*. A state-of-the-art case building located in Melbourne, Australia, housing advanced technologies, including ground source heat pump systems, is used to demonstrate and validate the research framework. A typical winter month in the southern hemisphere, July 2021, is monitored for the ground source heat pump systems designed and used for space heating. The findings reveal that the thermal energy generation during working days in July 2021 is close to the simulation results, with a difference of 2.2% in gross thermal energy production and a difference of 0.92% in inlet temperature. This research develops and validates an integrated approach for evaluating ground source heat pump systems, contributing to the utilisation of geothermal energy for building operations.

## Introduction

### Background

CO_2_ emissions from building operations increased to their highest level of around 10 GtCO_2_, or 28% of total energy-related CO_2_ emissions globally in 2019, which moved away from the Paris Agreement goal of keeping the global mean temperature rise to well below 2 °C^1^. Recent global trends and discussions related to climate change underscore the need to replace fossil fuels with renewable energy sources through existing and emerging technologies^2^. Many researchers have confirmed the enormous potential of geothermal energy both as a source of electricity and as a heating/cooling geosystem. Lund and Toth (2021) provided a worldwide review of the direct utilisation of geothermal energy, and concluded that the direct utilisation at the end of 2019 reached 107,727MWt, a 52.0% increase over the 2015 data^3^. Huang (2012) also explored geothermal energy in China, and concluded that the potential of generating power from geothermal energy in China is vast, but it is yet largely untapped^4^. Geothermal energy provides significant opportunities for high-performance buildings that require green energy for building operations. UNECE (2022) describes the role of high-performance buildings as ‘reducing the energy requirements of buildings to a point at which residual needs can be met by no or low-carbon energy sources’^5^. Renewable energy sources such as wind and solar have gained prominence in recent years. However, while geothermal energy is widely recognised as one of the most promising and ubiquitous renewable sources, the lack of performance evaluations publicly available hinders its application^6^. For example, although it has been identified that the geothermal potential in Australia is 26,000 times the nation’s annual primary energy consumption, the utilisation of geothermal is limited^7^. With the trends of subsidised geothermal energy installations^8^, it is worthwhile to investigate its potential for high-performance buildings.

**Figure 1:**
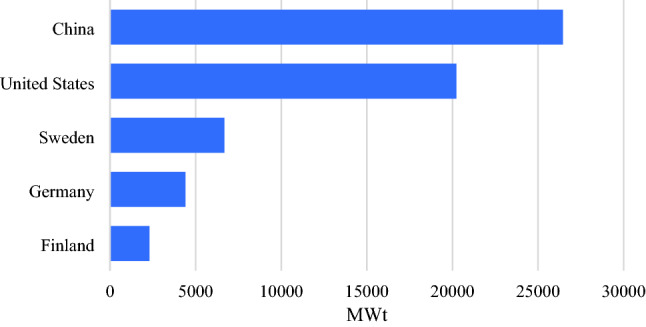
Worldwide leaders in the installation of GSHPs (refer to Lund and Toth^3^).

Geothermal energy can be deployed in different ways depending on the ground temperature: (1) for generating electricity when the temperature is above 150 °C; (2) for direct heating applications or indirect heating and cooling using heat pumps when the temperature is lower than 150 °C^9^. Among them, the use of heat pumps, namely Ground Source Heat Pumps (GSHPs), has increased significantly in recent decades worldwide due to their low carbon footprint and their ability to extract heat from the ground for building heating and cooling in different climatic typologies. This feature has increased the market interest in investing in GSHPs application and development as sustainable solutions. By 2020, the installed capacity of GSHPs has reached 77,547 MWt worldwide, with the leading countries highlighted in Fig. 1^3^. More importantly, previous studies confirmed that the efficiency of GSHPs is higher than that of other HVAC technologies^10^, with lower operational costs^11^. While the installation cost of GSHPs is still higher than traditional systems due to the high capital cost of GSHP units and installation through drilling or trenching^12^, they could be competitive solutions if additional incentives are offered by governments.

### Ground source heat pump systems

**Figure 2:**
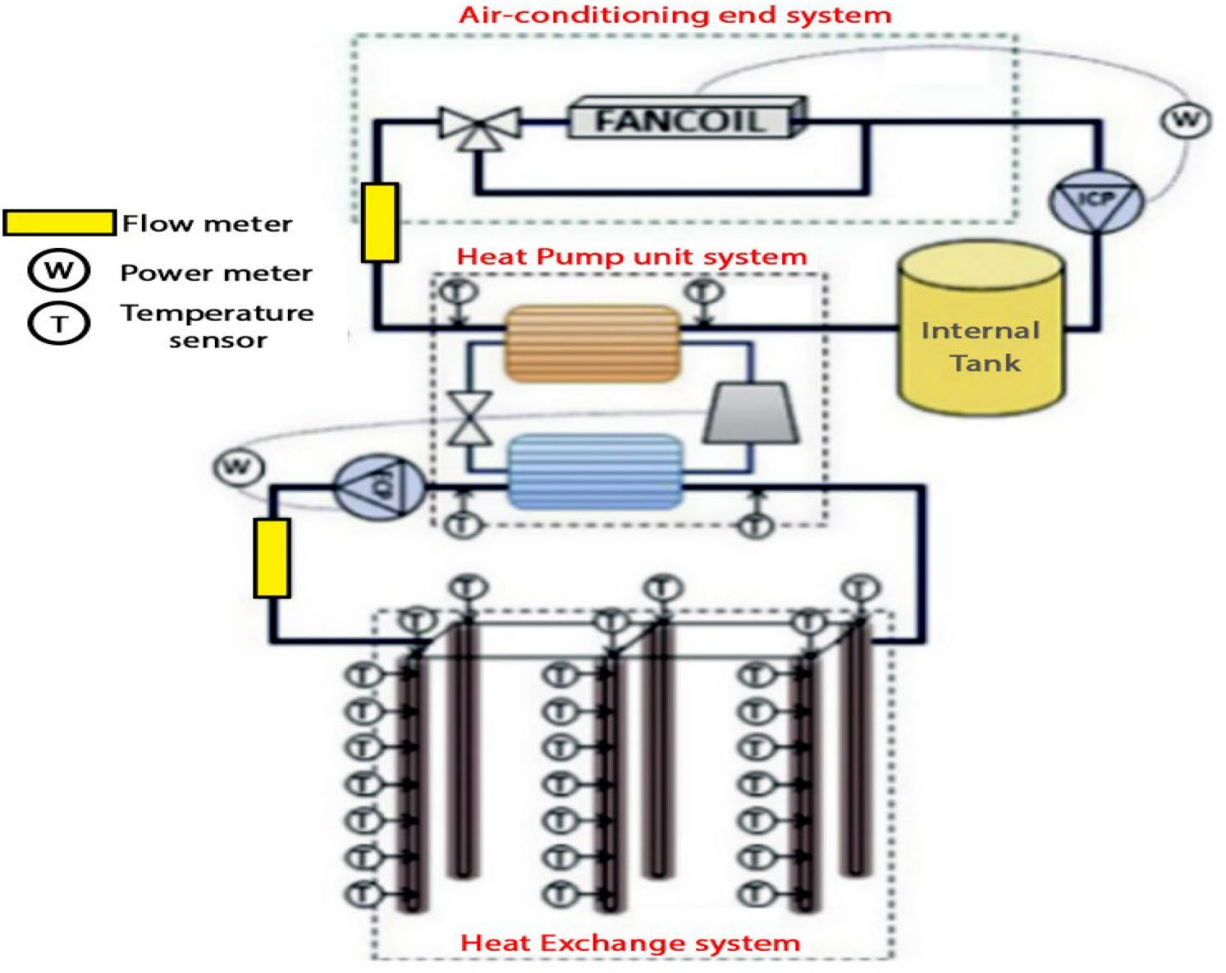
Schematics of a typical GSHP system (adopted from Ruiz-Calvo et al*.*^13^).

A GSHP system couples a heat pump with one or more ground heat exchangers (GHEs) in boreholes or trenches to transfer the ground thermal energy into the building. Figure 2 illustrates a general scheme of a GSHP system, which consists of three main components: (1) a geothermal heat pump unit, as the interface between indoor and outdoor sources, to modify the cycling medium temperature; (2) an air-conditioning end system that deals with building heating/cooling loads; and (3) heat exchange systems that are buried underground to facilitate heat extraction^13^.

**Figure 3:**
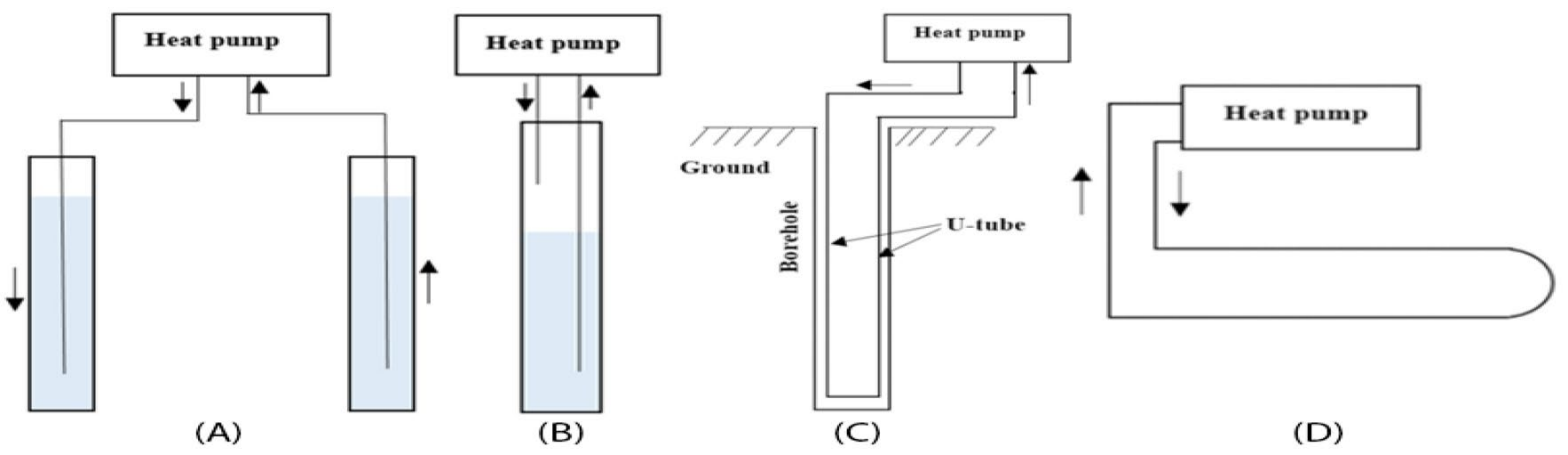
Geothermal heat exchanger types: open systems (**A**,**B**), and closed systems (**C**-Boreholes, **D**-Trenches) (adopted from Eswiasi & Mukhopadhyaya^14^).

There are two types of systems for implementing the GHEs: (1) open systems that use groundwater as the medium for heat exchange, in which there are no barriers between the groundwater and the soil (Fig. 3A,B); and (2) closed systems that, unlike open systems, employ a heat medium inside the heat exchangers, where the heat exchangers are separated from the soil by either vertical boreholes or horizontal trenches (Fig. 3C,D) containing high-density circulation pipes and a medium to carry heat (usually is water). Vertical GHEs outperform their horizontal counterparts for several reasons, such as less area required for installation and higher energy efficiency^14^, while the only disadvantage is the high initial cost of piping, borehole drilling, and refilling. Given the proven benefits, the most commonly-used mechanism is a closed vertical GHE (with a U-tube installed inside each borehole)^15^. It should be noted that, in cooling-dominated climates, heat pumps inject heat at higher temperatures into the soil in summer and cause temperature rises in the soil, which results in the cooling performance of GSHPs usually being lower than its heating performance^16^. In this regard, several studies have suggested coupling GSHPs with auxiliary energy source devices (hybrid GSHPs) such as cooling towers to overcome the temperature imbalance^17,18^.

### GSHP monitoring and guidelines

Research has suggested that it is vital to monitor the long-term performance to ensure the efficiency and reliability of GSHP systems and avoid potential problems, such as insufficient thermal energy^19^ and frozen pipes^20^. Although a number of experimental studies have been conducted worldwide, most of these studies have focused on the monitoring-based evaluation of the coefficient of performance (COP) of GSHPs^21,22^, where the overall COP of heat pumps is the ratio of the thermal energy output to the energy input required by the compressor. In addition to calculating the COP of GSHPs, a few studies have highlighted the existing challenges of GSHP metering and monitoring, such as incorrect temperature sensor installations, that can significantly affect the measuring and metering scheme^23^. To this end, Spitler et al. (2021) conducted a long-term monitoring study of a GSHP system performance as part of the international energy research and innovation program in buildings and communities, namely IEA-EBC Annex 52, towards net-zero energy buildings (NZEBs)^24^. This research focused on uncertainty analysis to understand the monitored measures and their associated errors due to the installed sensors. However, its application in simulation-based workflows is still unknown, and additional case studies with recent climatic changes are required to investigate the GSHP’s performance. Guidelines on monitoring the performance of GHSPs are also imperative. For example, the national standard in China (GB/T 50801-2013) evaluates the geothermal technology performance in three grades based on the energy efficiency of the entire system (EER) and suggests that monitoring should be performed a minimum of 15 days after the system becomes operational and maintained for at least 4 continuous days in order to obtain accurate results^25^. Notwithstanding these examples of national standards, each jurisdiction tends to apply different regulations for geothermal energy sources, and thus a common definition and framework has yet to be established^26^. As such, Lu et al*.*^21^, have concluded that there is an urgent need to develop a set of principles for the design and operation of GSHPs, as supplementary guidelines on the basis of existing industry standards, among which the guideline provided by the National Renewable Energy Laboratory (NREL)^27^ can be referred to for geothermal monitoring worldwide.

Therefore, there remains a lack of an integrated monitoring framework accompanied by overarching guidelines. Given the lack of an integrated monitoring framework, underscored by the literature review above, the development of an integrated monitoring framework accompanied by an overarching guideline for investigating GSHP systems is imperative.

### GSHP simulation

The importance of performance simulation during the design stage of a project is already well established, and the absence of such tools would pose significant challenges in efforts to design sophisticated, energy-efficient, and environmentally-friendly buildings^28^. In recent decades, many studies have investigated the potential of GSHPs using software modelling and artificial intelligence to predict performance and obtain optimal technical configurations^29,30^. Soil properties, building loads, and meteorological data have been found to be the main factors influencing the design and performance of GSHP systems^31^, while the heat transfer calculation process in GHEs was found to be challenging due to uncertain factors, such as ground thermal properties and groundwater flow rate, over a period^32^. However, most of the available simulation programs, such as TRNSYS (a graphics-based software environment used to simulate the behaviour of transient systems, such as thermal and electrical energy systems) and EnergyPlus (a whole building energy simulation program used to model energy performance), are not sufficiently developed to accurately capture the ground thermal performance (e.g., temperature distribution or heat capacity) or the interaction between GHEs, especially during operation^33,34^. Cimmimo (2018) has noted that, while GSHP simulations intended to predict the returning fluid temperatures from the boreholes as well as the ground temperatures in the bore field, failure to predict the temperatures accurately would compromise the accuracy of any subsequent GSHP performance analysis and system design efforts, especially in the case of horizontal GHEs, where they are more subject to ground surface temperatures due to sun exposure and ambient conditions^35^.

Accordingly, numerical design methods have been proposed to model and simulate the heat exchanger response for the purpose of predicting ground temperatures from specific bore field configurations. The most widely used method, developed by Eskilson, is called ‘g-function’^36^. G-function is also referred to as a thermal response factor that allows for accurate dynamic temperature calculations of the fluid and ground during the design period, facilitating the prediction of the GSHP system performance and components’ sizing^37^. This method was used by the American Society of Heating, Refrigerating and Air-Conditioning Engineers (ASHRAE) direct design method for modelling^38^, and several studies have implemented g-functions as model inputs to building simulation tools to predict GSHP and building energy performance on an annual or hourly basis^39,40^. Other EnergyPlus-based applications using g-functions for modelling the bore field performance of the GSHP system include GLHEPro and Earth Energy Designer (EED), and even the official DesignBuilder documentation recommends computing g-functions externally and adjusting them according to the simulation procedure^41^.

However, the g-function method is limited by several assumptions that may affect its accuracy, including (1) that the boreholes in the bore field are of equal dimension (i.e., length and radius), (2) that the boreholes are connected in parallel, with no series interconnections, and (3) that the boreholes in the field are uniformly spaced^42,43^. In most practical systems, these assumptions do not hold, meaning that the computed g-functions may be inaccurate, and this can have a significant impact on the long-term ground temperature simulation. Cimmino and Bernier thus presented a method to compute g-functions for boreholes connected in any configuration of series and parallel connections^44^. The modified g-function method is available to use as a Python-based package^45^.

### Research objectives

As underscored by the literature review, geothermal energy could potentially be a great source for high-performance buildings, with less carbon footprint and lower operational costs. However, an integrated framework for evaluating GSHP systems through performance monitoring and simulation has yet to be established. Furthermore, the incapability of well-known simulation tools in accurately capturing ground thermal performance also hinders its application. Therefore, this paper proposes an integrated framework for evaluating the performance of GSHP systems, unlocking geothermal energy utilisation for high-performance buildings. The objectives underlying the research presented in this paper are to:

- Improve and amend the NREL guideline for the monitoring of geothermal energy;
- Develop a sensor-based monitoring framework for evaluating the actual energy performance of GSHP systems, aligning with the monitoring guideline; and
- Identify and validate a ubiquitous and effective approach for the energy simulation of GSHP systems based on monitored data.

A state-of-the-art building located in Melbourne, Australia, is used as a case study to demonstrate the proposed framework, where the uniqueness of the geothermal application provides opportunities for research innovation in this paper. This research is to unlock GSHP utilisation through an integrated framework, including an overarching improved monitoring guideline, a monitoring prototype and instrument, and a g-function-based simulation approach, thereby advancing the utilisation of geothermal energy in high-performance buildings worldwide.

## Methods

**Figure 4:**
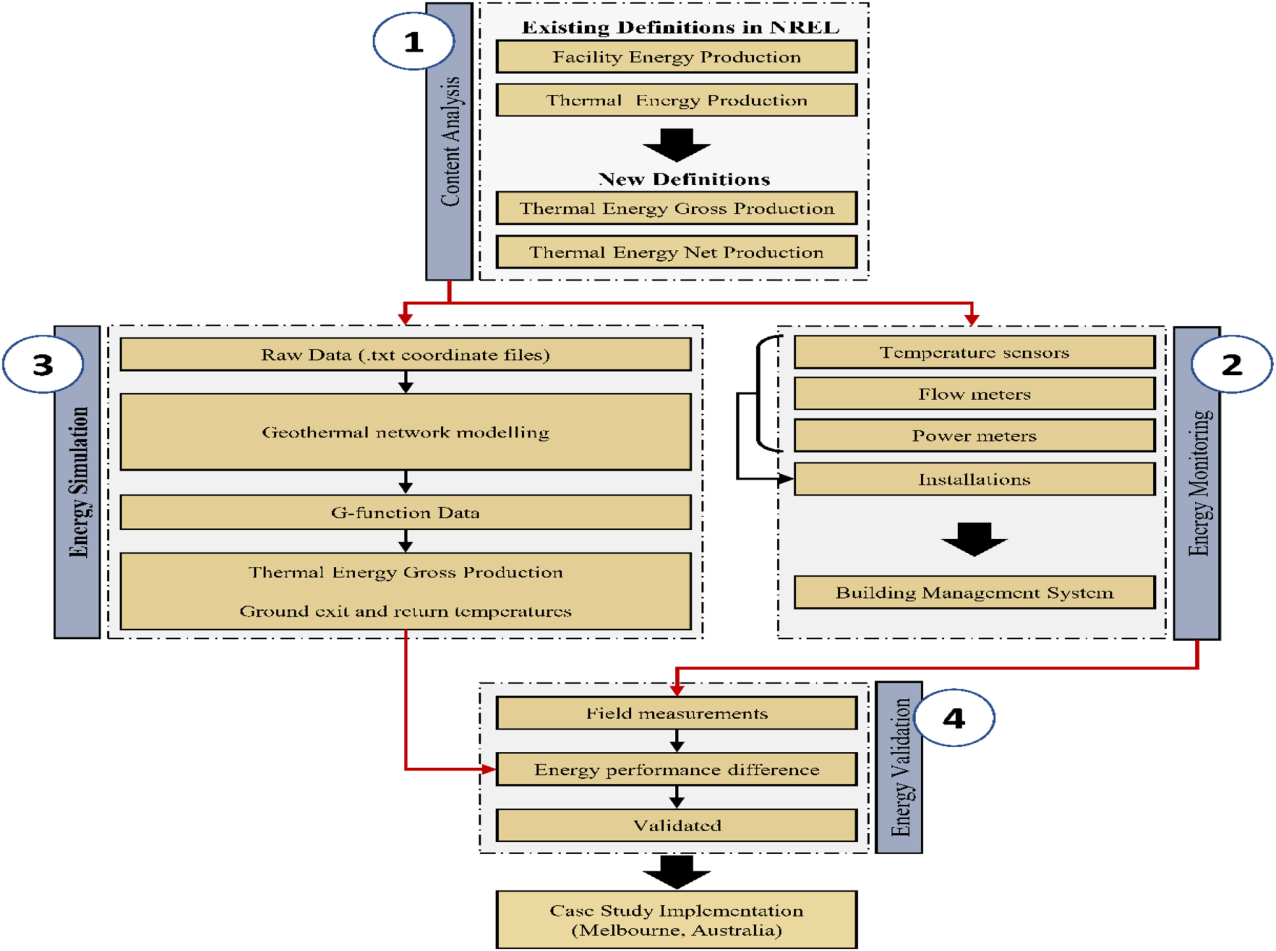
Research methodological framework and process.

The methodology underlying this research comprises four interconnected pillars, as illustrated in Fig. 4: (1) the first pillar analyses the US NREL guideline and provides the rationale for the improvement based on the analysis, (2) the second pillar outlines the monitoring framework and metrics for monitoring GSHP systems, (3) the third pillar covers the simulation-based workflow performed in this research, and (4) the fourth pillar validates the simulation results against field measurements collected in the second pillar, based on the developed simulation-based method in a real case building in Melbourne, Australia.

### Content analysis

Appropriate guidelines are imperative for monitoring the performance of GSHP systems. As discussed in the literature, the NREL guideline in the U.S. is the closest one to what could be considered a universal guide for GSHP system applications and particularly for measuring building energy performance^42^. The NREL guideline categorises geothermal energy generation as part of **Facility Energy Production,** which is defined as the ‘Total of all energy produced at the facility and either used at the facility or sold for use elsewhere, excluding losses within the energy production systems. [It] includes Thermal Energy Production and electrical energy produced by PV, wind, geothermal, or other means, minus Electrical Generation System Losses.’ **Thermal Energy Production**, meanwhile, is defined as the ‘Thermal energy generated at the facility by means such as solar thermal or geothermal, to the extent that the energy is used at the facility in a way that offsets the consumption of purchased energy or other energy generated at the facility.’ However, the monitoring protocol is still not sufficiently explained in the NREL guideline. For example, it does not address (1) whether the thermal energy production of GSHP systems should be measured at the inlets or outlets of GSHPs, despite the fact that this is an important consideration in characterising the relationship between the thermal energy production and electricity consumption by GSHPs; or (2) what should be measured for the purpose of assessing the thermal energy production if the electricity consumption by GSHPs is not monitored separately. Therefore, a number of amendments and improvements to the guideline for the energy monitoring of GSHP systems are proposed, as described below.

**Figure 5:**
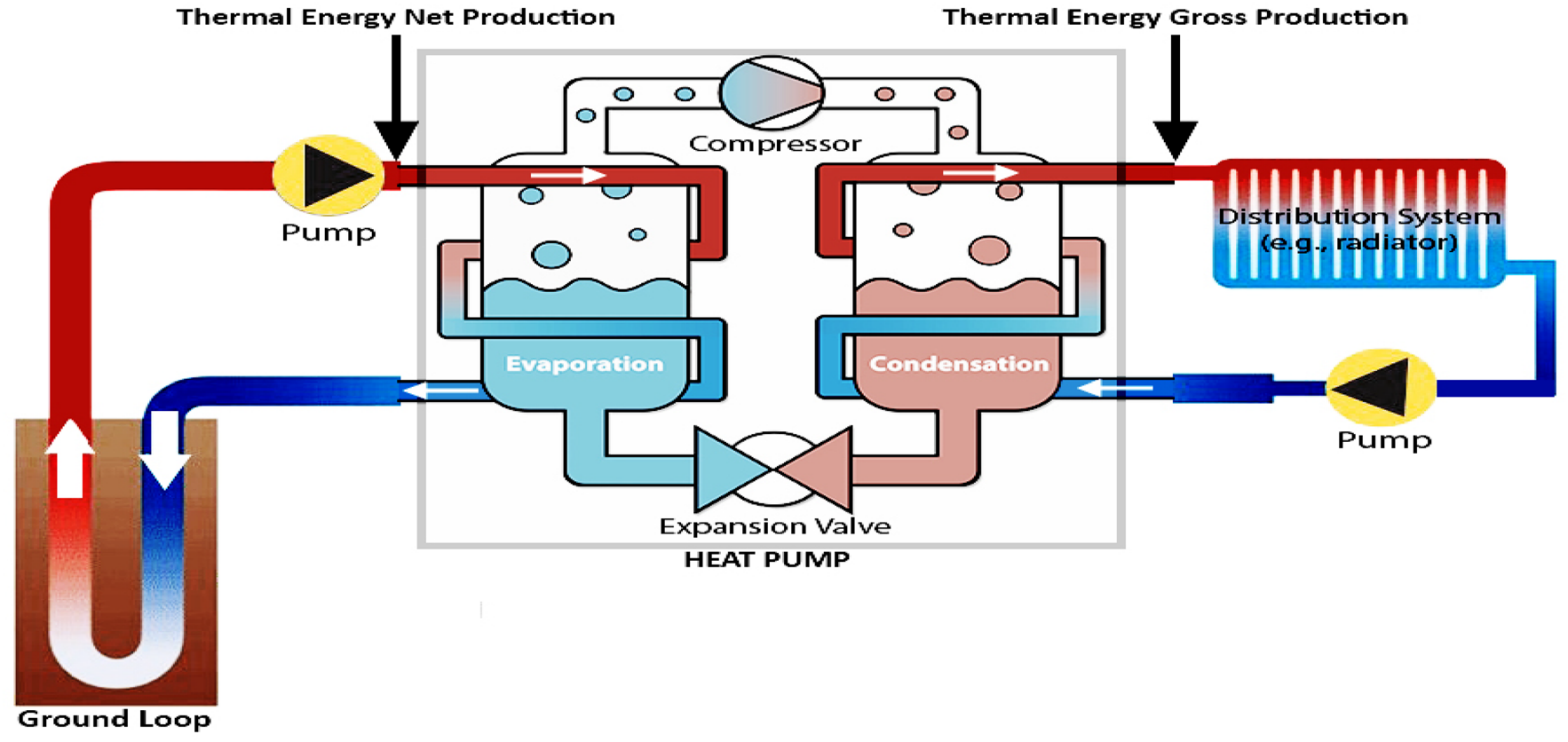
Thermal energy net and gross productions in GSHPs.

For the purpose of this research, it should be noted, ***Thermal Energy Gross Production*** is defined as the overall thermal energy generation that can be directly used for building operation, and it can be monitored at the outlet points of GSHPs, as illustrated in Fig. 5. Based on the first law of thermodynamics, the energy used by the GSHP also contributes to the gross generation through energy conversion. Therefore, ***Thermal Energy Net Production*** is defined as *Thermal Energy Gross Production* minus the *electrical consumption of geothermal heat pumps*, and it can be monitored at the inlet points of GSHPs, as illustrated in Fig. 5. *Thermal Energy Net Production, Thermal Energy Gross Production,* and *electrical consumption of GSHPs* have the following relationship (Eq. 1).

1 $$Thermal\;Energy\;Net\;Production = Thermal\;Energy\;Gross\;Production - Electrical\;Consumption\;of\;GSHPs$$

As such, when there are dedicated submeters for *Thermal Energy Gross Production* and the *electrical consumption of GSHPs*, *Thermal Energy Net Production* can be derived using Eq. (1). Similarly, when there are dedicated submeters for *Thermal Energy Net Production* and the electrical consumption of GSHPs, *Thermal Energy Gross Production* can also be derived using Eq. (1). However, in most buildings, there are no dedicated submeters to monitor the electrical consumption of a GSHP individually, and instead, the electrical consumption of a GSHP is usually monitored together with other components. In this scenario, the thermal energy generation can only be monitored by positioning thermal meters at the inlet points of GSHPs to monitor the *Thermal Energy Net Production*. Otherwise, the thermal energy generation will be overcounted by incorporating the energy conversion of electrical consumption of GSHPs.

### Performance monitoring

To evaluate the actual performance, an intelligent monitoring prototype for GSHP systems that align with the guidelines available must be developed. Compared to those for other renewable energy systems, such as solar PV, the monitoring systems for geothermal technologies are less developed and need to be customised for specific buildings. Therefore, a monitoring prototype that uses thermal meters and temperature sensors, along with data loggers or a Building Management System (BMS), is proposed for GSHP systems in this research. The actual energy generation can be acquired from the thermal meters.

In the NREL guideline, the gross energy generation from GSHPs is used as the indicator of energy generation; however, based on the first law of thermodynamics, the energy used by the GSHP also contributes to the gross generation through energy conversion. Therefore, the monitoring prototype proposed in this research incorporates key considerations for the instrument design and installation, including (1) measurement metrics, i.e., what should be measured to achieve the research objectives; and (2) installation locations, i.e., where to install the instrument to monitor the performance, based on monitoring objectives and in alignment with the improved monitoring guideline. Moreover, in this research, both thermal energy and temperature are evaluated and validated, with the instrument locations aligned with the simulation outputs for validation purposes. In this regard, thermal meters are installed at the outlet points of the supply side of the GSHP systems in order to monitor the *Thermal Energy Gross Production*. Additional temperature sensors are also installed at the inlet points of the source side of the GSHP systems to monitor the temperature of water circulated from underground, representing the thermal response of the water loops.

### Performance simulation

To address the identified research gap, third-party g-function computations for GHE modelling are conducted; the results are then integrated into the building simulation tool, DesignBuilder, for the subsequent energy performance analysis of GSHP systems. This research will also examine the impact of independently computed g-function data on the energy performance simulations/analysis, this being achieved through comparison and validation with the monitored results.

In this research, the g-function approach proposed by Cimmino (2014) is used to simulate the thermal response of the boreholes, based on effective borehole wall temperature^44^. Effective borehole wall temperature is defined based on inlet fluid temperature and effective bore field thermal resistance. These g-functions are then superimposed in time to obtain the effective borehole wall temperature variation due to variable heat extraction rate in the bore field as per the expression below^37^:

2 $$T_{b}^{*} \left( t \right) = T_{0} - \frac{1}{{2\pi k_{s} }}\mathop \smallint \limits_{0}^{t} \overline{Q}^{^{\prime}} \left( {t - t^{\prime}} \right)\frac{dg}{{dt}}\left( {t^{\prime}} \right)dt^{\prime}$$

where $T_{b}^{*}$ represents the effective borehole wall temperature, $T_{0}$ indicates the undisturbed ground temperature, $\overline{Q}^{^{\prime}}$ denotes the average heat extraction rate per unit borehole length, and $g$ represents the g-function of the bore field. The effective borehole wall temperature is related to the mean fluid temperature in the bore field through the bore field thermal resistance as expressed below^37^:

3 $$\overline{{T_{f} }} \left( t \right) = T_{b}^{*} \left( t \right) - R_{field}^{*} \overline{Q}{^{\prime}}\left( t \right)$$

where $\overline{{{\text{T}}_{{\text{f}}} }} = 0.5\left( {{\text{T}}_{{{\text{f}},{\text{in}}}} + {\text{T}}_{{{\text{f}},{\text{out}}}} } \right)$ represents the arithmetic mean of the inlet and outlet fluid temperature in the bore field, and $R_{field}^{*}$ represents the effective bore field thermal resistance.

A Python-based platform, Pygfunction, is then employed to calculate g-functions^45^. Pygfunction, it should be noted, is a Python module used for the modelling of geothermal bore fields and subsequent computation of the thermal response factors, or g-functions, for these borehole fields^46^. It is based on the analytical finite line source solution for the evaluation of the thermal interference between boreholes in the same bore field, which allows for the rapid calculation of g-functions. Pygfunction can be used to predict variations in borehole temperature and in the evaluation of borehole fluid temperatures. The notable feature of Pygfunction absent in comparable tools is that it allows for the calculation of interconnected boreholes of different lengths and radius.

In this respect, the first step in computing the g-function for any given bore field using Pygfunction is to create the model representation of the bore field using the Pygfunction *Boreholes* and *Networks* modules. However, to model precise borehole placements, a correctly formatted .txt file containing the coordinates, depth, and radius is required. The borehole coordinate data can be extracted from the AutoCAD (or other drafting software) drawings for the bore field and used to populate a formatted text (.txt) file. For this purpose, the *Boreholes* module in the Pygfunction contains information regarding the dimensions and positions (coordinates) of boreholes. Several different example Python scripts can be run for the modelling of regular bore field shapes, including L-shaped, U-shaped, and circular bore field configurations.

Series interconnections among the boreholes in the field also need to be modelled in the *Networks module* within Pygfunction, which is a critical feature of Pygfunction compared with other tools. This module is used to model the series, parallel, and mixed networks/interconnections between boreholes in the field. Ensuring that the network layout is simulated exactly as-built is paramount to obtaining an accurately computed g-function, and this function represents a key difference between Pygfunction and other modelling and computational methods, which are limited in terms of their capacity to handle complexities within the borehole interconnections and loops. The innovative methodology proposed in this research endeavours to fill this largely unaddressed gap.

In the *Networks module* within Pygfunction, the ‘bore_connectivity’ variable is used for the creation of the networked model. The variable can be created using the following rules, as outlined in the instructions: “*Index of the fluid inlet into each borehole where − 1 represents a borehole connected to the bore field inlet. If this parameter is not provided, parallel connections between boreholes are used. Default is None.”* For example, for a bore field with 6 boreholes (labelled 0 to 5) connected in two circuits (0–1–2 and 3–4–5), the result would be: bore_connectivity = [− 1, 0, 1, − 1, 3, 4], where − 1 indicates that the borehole is connected to the field inlet. The borehole model and networked model created can also be visualised using Python scripts.

After creating the bore field model based on the Pygfunction, the next step is to perform the g-function computations for the given bore field configuration. The *g-function module* within Pygfunction is used for this purpose. The configuration and properties of the boreholes in the field are defined using the *Boreholes module*, and the *g-function module* is then used to compute the g-function. G-functions, it should be noted, can be computed for fields with equal inlet fluid temperature conditions, fields with mixed inlet fluid temperatures, uniform heat extraction along boreholes, and uniform borehole wall temperature. For mixed inlet fluid temperatures due to the series connections between the boreholes in each circuit or network, the following parameters are used to calculate the g-function for a bore field: (1) list of boreholes included in the bore field; (2) inlet mass flow rate into each circuit of the network, *m_flow* (kg/m^2^); (3) fluid specific isobaric heat capacity, *cp* (J/kg°C); and (4) soil thermal diffusivity, *alpha* (m^2^/s).

Finally, the computed g-function is imported to a simulation tool that accommodates g-functions for the simulation of GSHP systems. An EnergyPlus-based software, DesignBuilder, is one of the tools that use g-functions for the energy simulation of GSHP systems. A wide range of heat pumps is made available for users to modify the heating and cooling components, flow rate, heating capacity, heating compressor power coefficients, and so on. Moreover, the GHE component data is modelled through a simple dialogue box as part of the DesignBuilder HVAC system. Meanwhile, the computed g-function data is imported into the DesignBuilder program to carry out the energy performance analysis and simulate ground inlet/outlet temperatures.

### Performance validation

**Figure 6:**
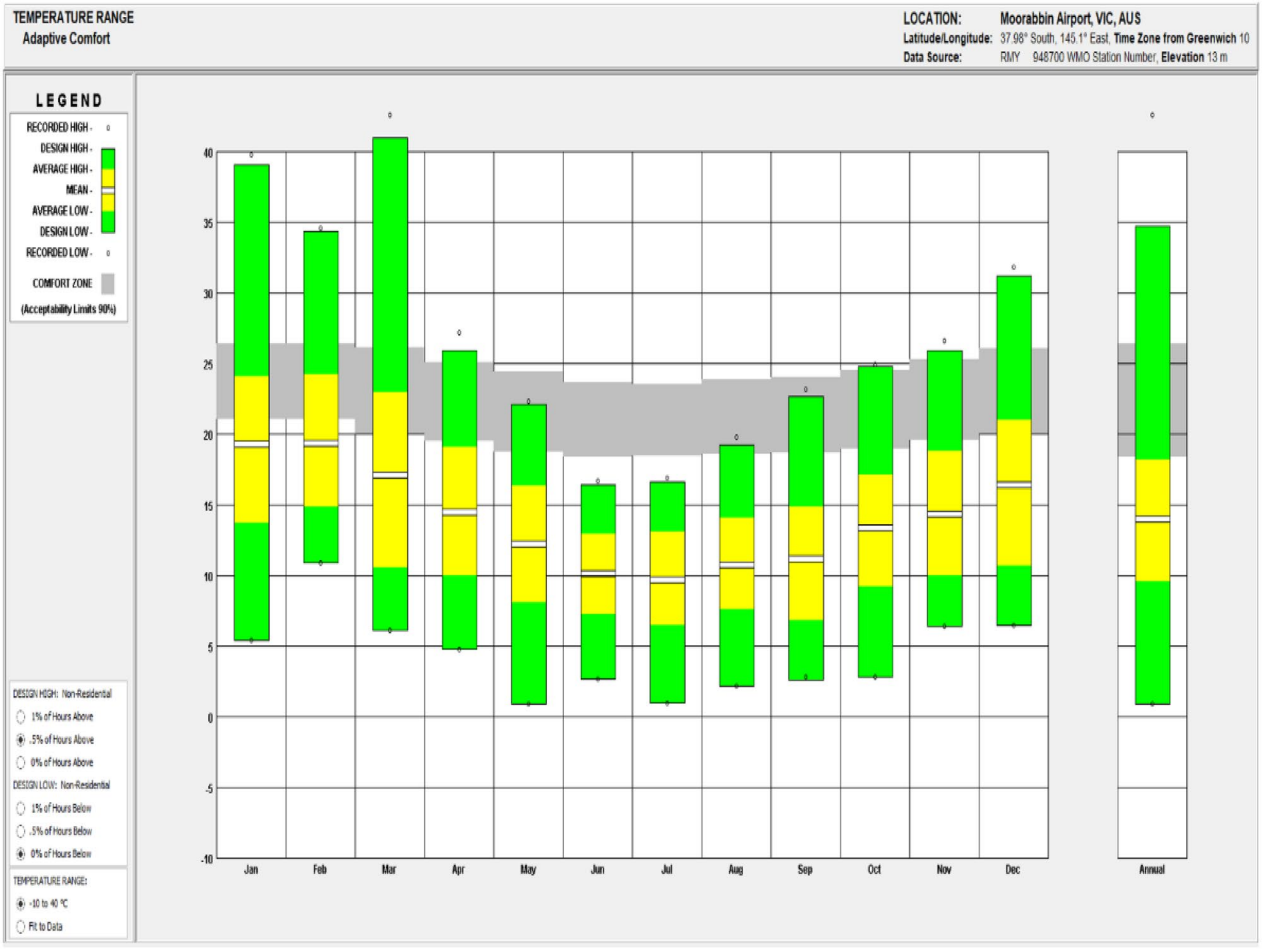
Average temperature of site location.

**Figure 7:**
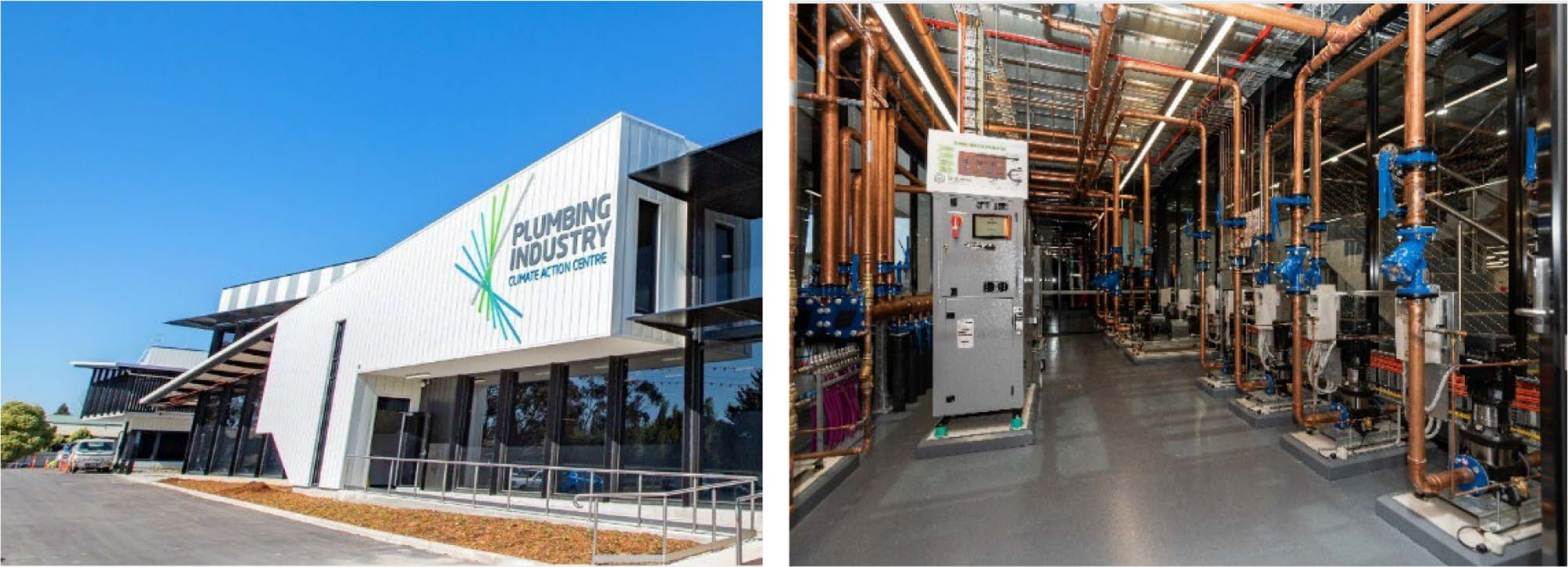
Case building and plant room.

As part of the validation procedure, the thermal energy gross production and the inlet temperature of the source side of the GSHP systems are simulated using DesignBuilder, and these are then compared with the field measurements outlined in “Performance monitoring” section. Validation of the simulation approach provides a solid foundation for the implementation of the framework proposed in this research. In this respect, a leading zero energy building (ZEB) in Melbourne is selected as a case study. The average temperature of this building location is illustrated for each month in Fig. 6. A ZEB, it should be noted, is defined as a building generating as much energy from renewable sources as it consumes on an annual basis and requiring zero energy from fossil fuels on an annual basis during operation. The case building uses a combination of renewable technologies, including geothermal, solar PV, and solar thermal, to achieve the zero-energy building goal, and it is the first building in Australia to use building foundation screw piles for geothermal energy generation. Other technologies used in this building include in-slab hydronic heating, packaged air conditioning (PAC) units, fan coil units, and evaporative cooling. There are two GSHPs connected to the geothermal ground heat exchangers extracting thermal energy for building heating purposes. The case building and the geothermal plant room are illustrated in Fig. 7. The uniqueness of the geothermal application in this climate provides opportunities for research innovation, as described in this paper. Thus, the developed research framework is applied to this case building in order to better understand and advance geothermal energy utilisation, including through performance monitoring and energy simulation and the proposed monitoring guideline improvements.

## Case study and results

**Figure 8:**
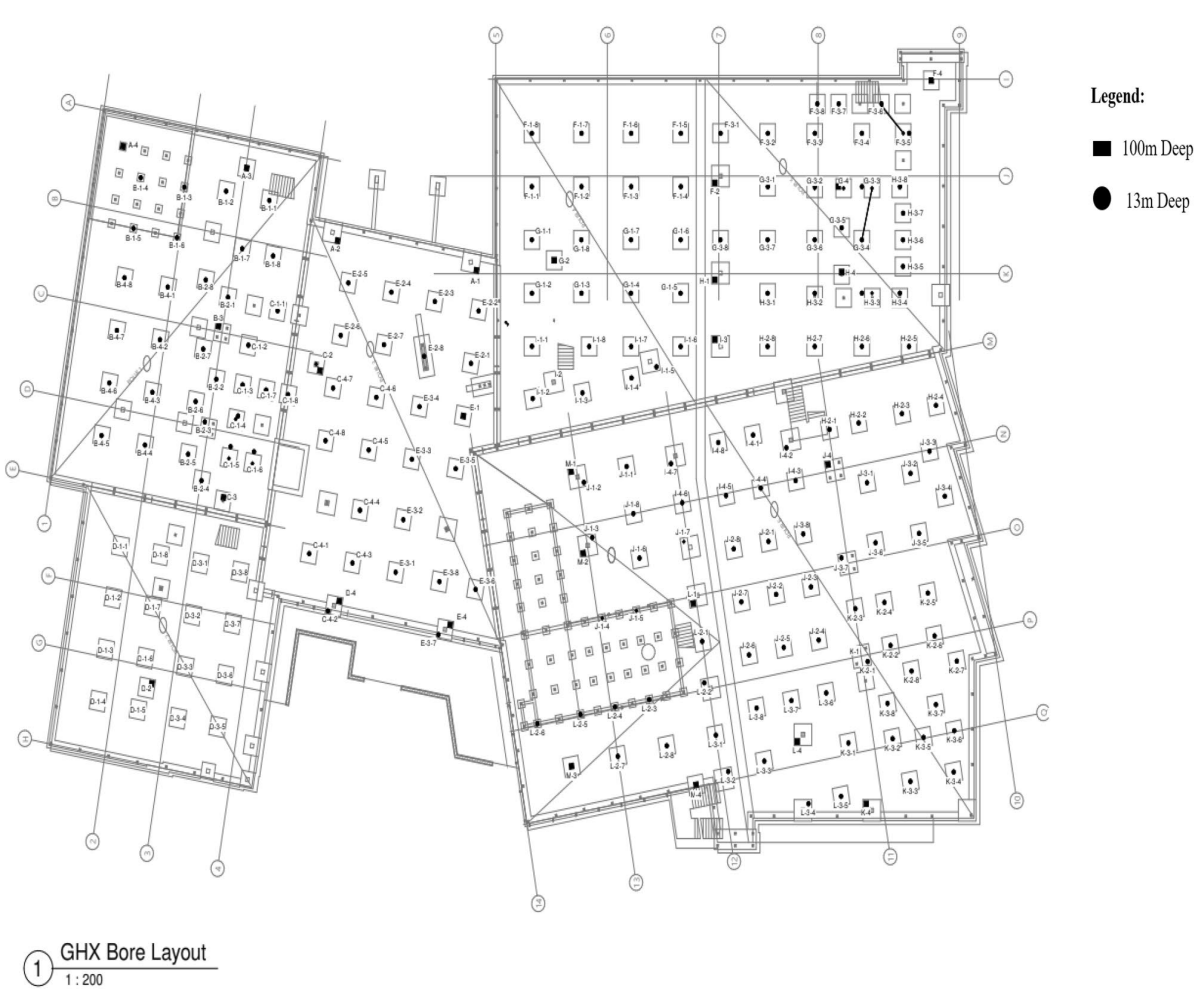
Geothermal bore field layout.

The geothermal bore field comprises 220 boreholes integrated with ground heat exchangers, installed at two different depths (192 boreholes installed at a depth of 13 m below ground, and 28 boreholes installed at a depth of 100 m below ground). The borehole layout for the building is shown in Fig. 8. The 220 ground heat exchangers are built into 13 circuits, with each circuit featuring several boreholes interconnected serially, resulting in mixed inlet fluid temperatures due to the series connections between the boreholes in each circuit or network.

### Performance simulation

**Figure 9:**
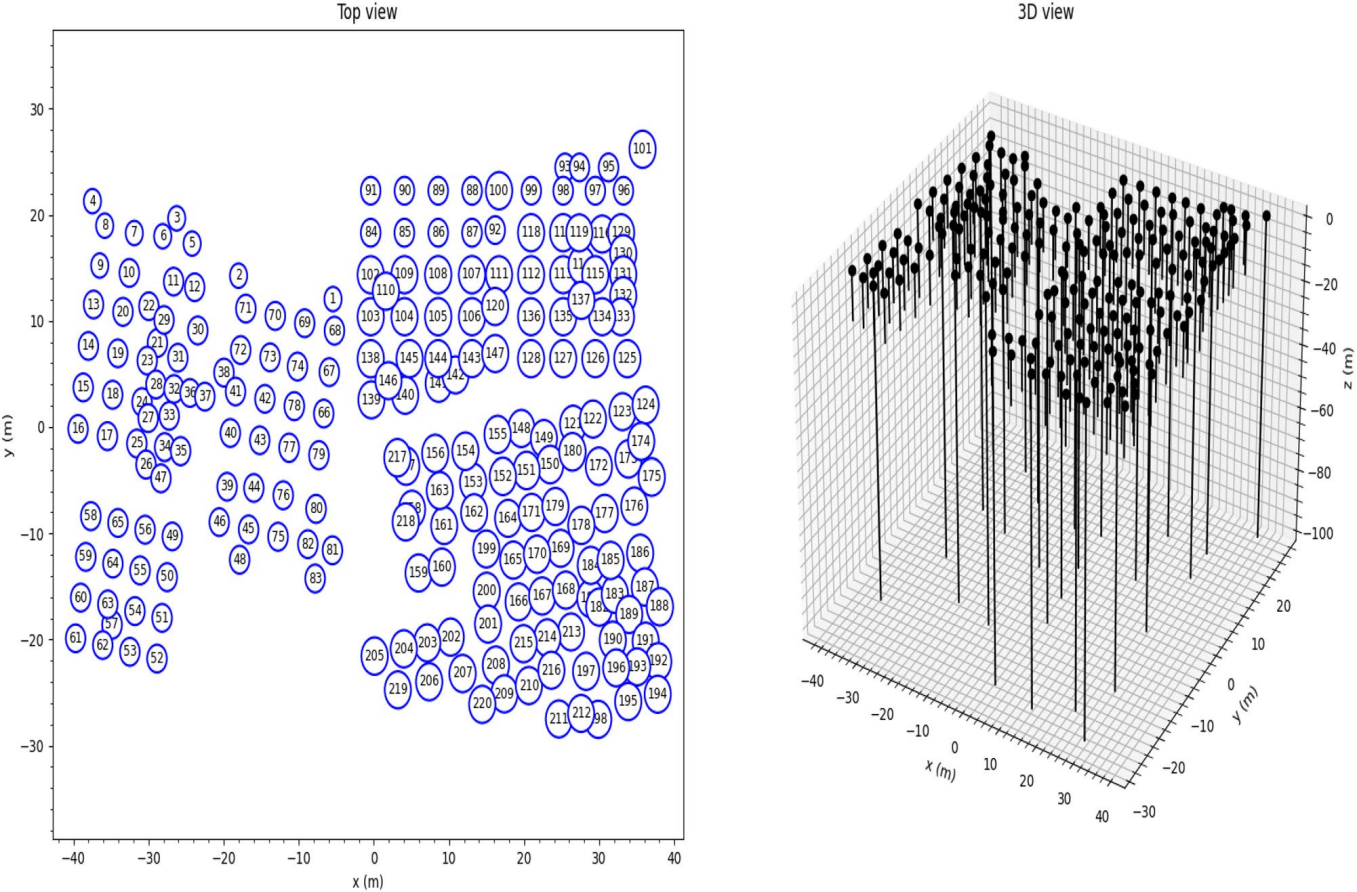
Modelled building geothermal bore field with Pygfunction.

**Figure 10:**
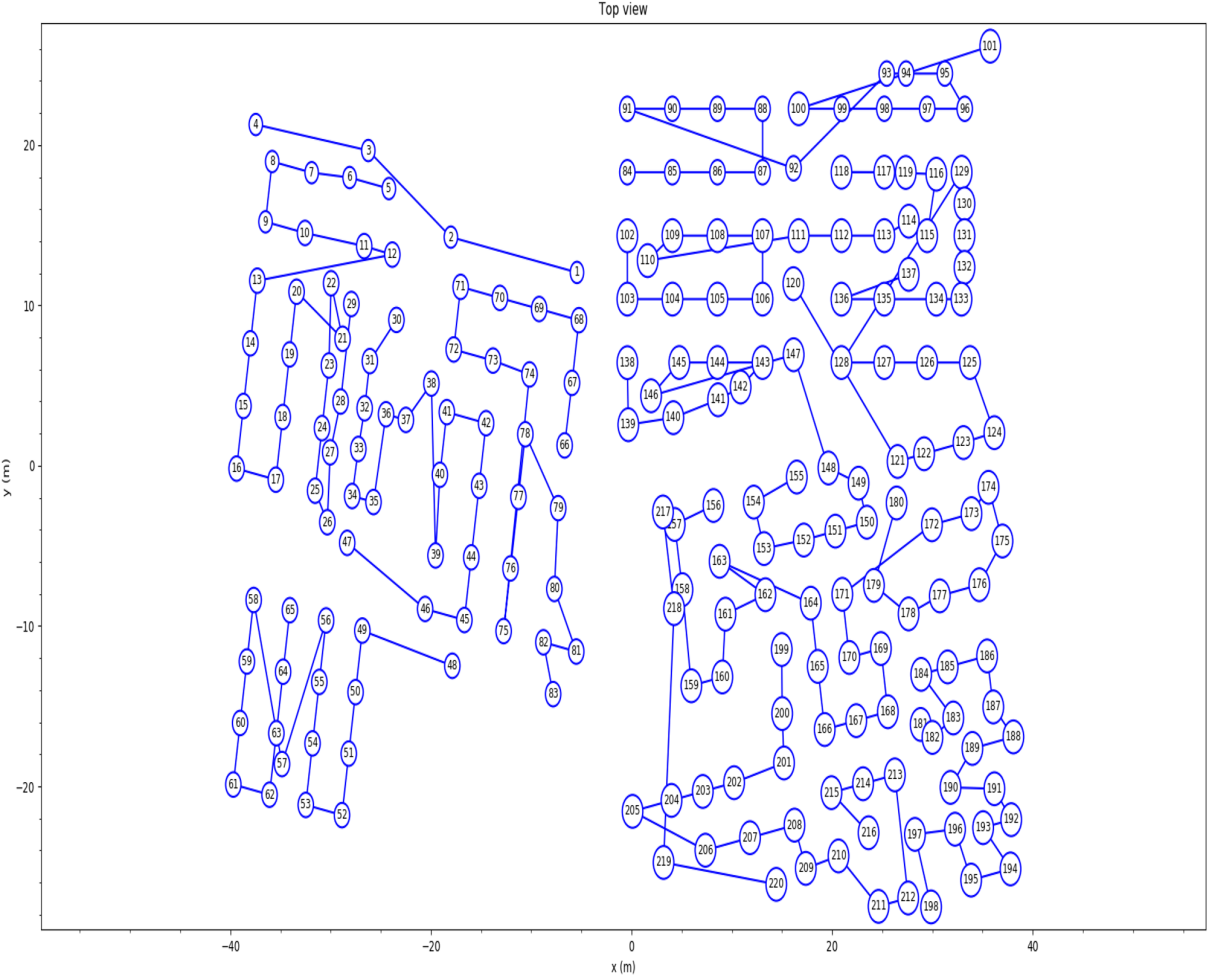
Bore field network layout.

**Figure 11:**
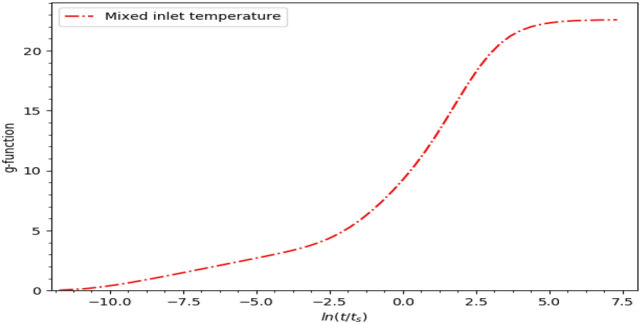
Graphical view of the case building g-function.

The geothermal bore field modelled in Pygfunction is illustrated in Fig. 9, while the bore field network layout is displayed in Fig. 10. Based on the parameters required for mixed inlet fluid temperatures, the Python script is used to calculate the g-function for the case building, with the resulting g-functions presented in Fig. 11.

Finally, for the purpose of performance analysis using the EnergyPlus-based program, DesignBuilder V7^45^, the g-function data computed using Pygfunction is imported into the program. The input data file (IDF) editor in EnergyPlus can be used for this purpose, or a simple copy-paste command can be used to transfer the computed g-function. Based on the computed g-function, the thermal energy generation and the inlet temperature of the GSHPs at the supply side, which reflect the thermal response of water loops from underground, are simulated.

### Performance monitoring

To evaluate the actual performance, the developed monitoring instrument is installed in the case building in accordance with the improved guideline. To achieve the research objectives for this case building, both thermal energy and temperature are measured for evaluation and validation purposes. Based on the proposed monitoring framework, a customised monitoring instrument is designed and installed for the case building, with the key considerations for the instrument design and installation in this research including: (1) measurement metrics, i.e., ***gross thermal energy production***, and the inlet temperature of the GSHPs; and (2) installation locations, these being determined in accordance with the improved monitoring guideline and the simulation outputs for validation purposes.

**Figure 12:**
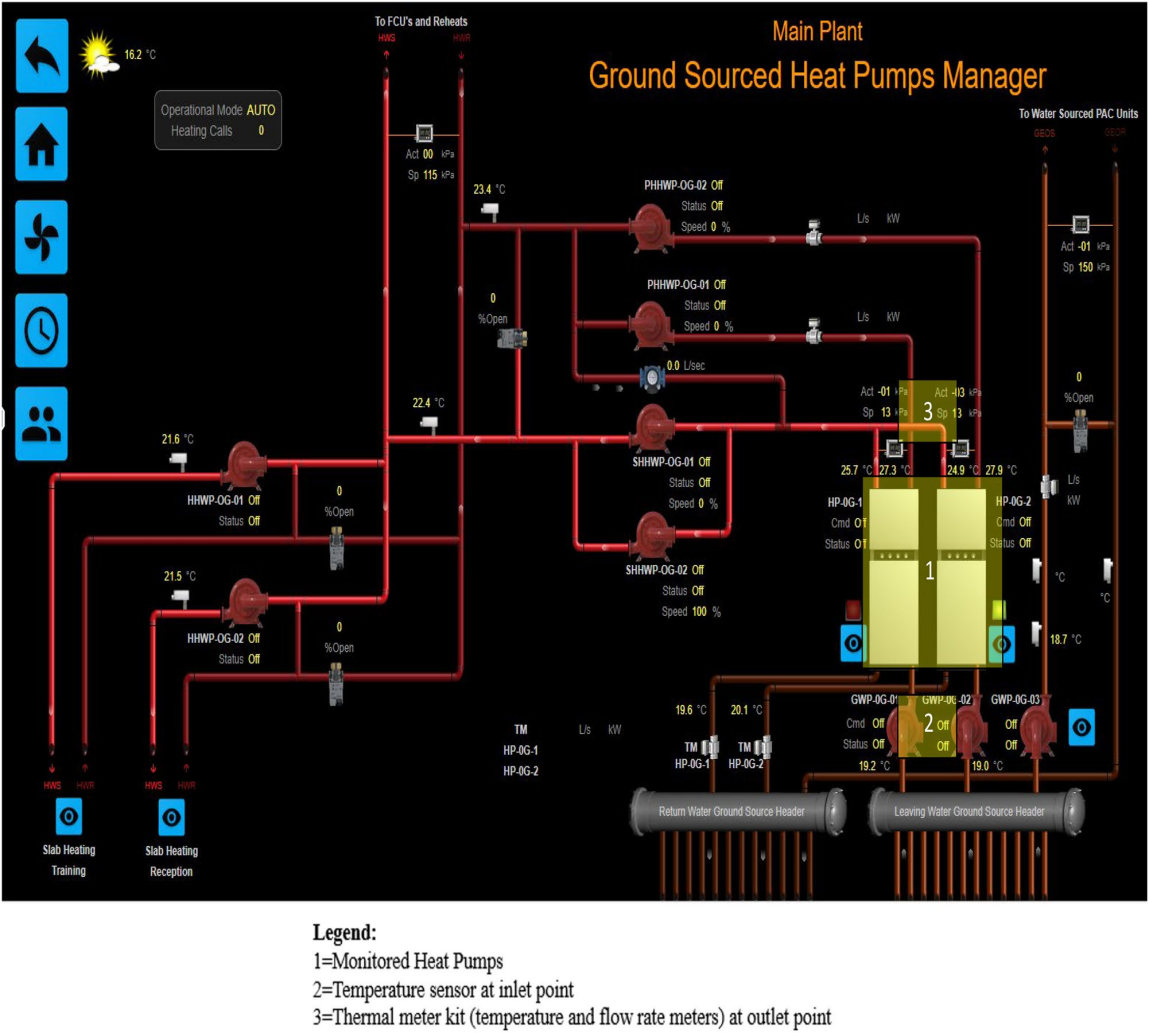
Schematic diagram of the GSHP system.

In this research, thermal meter kits are installed at the outlet points of the supply side of the GSHPs to monitor the ***gross thermal energy*** generated. Additional temperature sensors are installed at the inlet points of the source side of the GSHPs to monitor the water temperature circulated from underground, as this represents the thermal response of the water loops. The installation locations are illustrated in Fig. 12. The monitored data is stored in the BMS, where it is readily accessible and downloadable. The collected data is used for energy performance evaluation and validation against the simulation results, as described below.

### Comparison and validation

**Figure 13:**
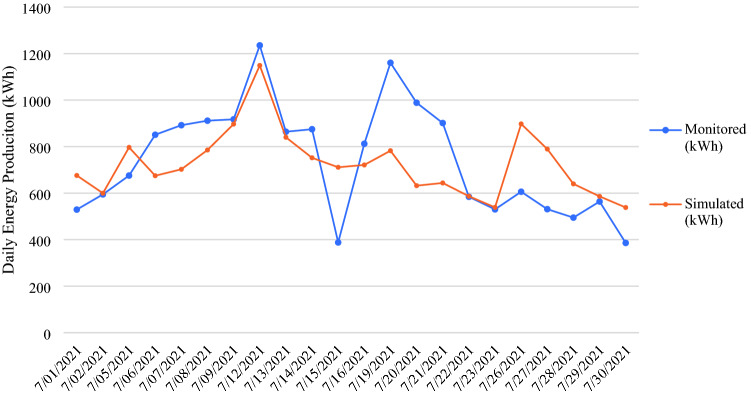
Monitored and simulated thermal energy production.

Given that the two GSHPs are used for space heating in the winter season but then are switched off for most of the summer season, the data collected during a typical winter month, July, is used for analysis and validation in this research. The gross thermal energy generated from the monitored GSHPs *during working days* in July 2021 is listed in Table 1 and plotted in Fig. 13. The overall difference between the monitored and simulated energy production is 2.2%, with the Mean Absolute Deviation (MAD) of 150.3.

**Table 1:** Monitored and simulated thermal energy production and comparison.

Date	Working days	Monitored (kWh)	Simulated (kWh)	Absolute Value of Error
7/1/2021	Thursday	529.2	676.0	146.8
7/2/2021	Friday	594.6	599.7	5.1
7/5/2021	Monday	675.6	796.8	121.2
7/6/2021	Tuesday	851.0	674.8	176.2
7/7/2021	Wednesday	892.1	702.5	189.6
7/8/2021	Thursday	911.5	785.4	126.1
7/9/2021	Friday	917.3	896.9	20.4
7/12/2021	Monday	1235.4	1149.0	86.4
7/13/2021	Tuesday	864.0	840.1	23.9
7/14/2021	Wednesday	874.8	751.9	122.9
7/15/2021	Thursday	388.0	711.2	323.2
7/16/2021	Friday	812.3	721.2	91.1
7/19/2021	Monday	1160.5	782.4	378.1
7/20/2021	Tuesday	988.7	632.1	356.6
7/21/2021	Wednesday	901.4	643.7	257.7
7/22/2021	Thursday	584.3	586.7	2.4
7/23/2021	Friday	530.2	538.8	8.6
7/26/2021	Monday	606.0	897.6	291.6
7/27/2021	Tuesday	531.1	789.5	258.4
7/28/2021	Wednesday	494.7	639.8	145.1
7/29/2021	Thursday	563.9	586.1	22.2
7/30/2021	Friday	385.9	538.3	152.4
Total		16,292.5	15,940.6	3306.0
Difference	(Monitored-Simulated)	351.9 kWh		
	(Monitored-Simulated)/Monitored	2.2%		
Mean Absolute Deviation (MAD)	(Total Absolute Value of Error)/N			150.3

The hourly inlet temperature of the GSHPs was monitored for the same month, i.e., July 2021. The average simulated temperature based on the computed g-function for July is found to be 15.14 °C, while the average monitored temperature is 15.00 °C, resulting in a difference of 0.92% in inlet temperature. The results and comparison between the simulated and monitored data demonstrate and support the efficacy of the overall framework proposed in this research.

## Discussion

Building operations have increased to their highest level at around 10 GtCO_2_, or 28% of total global energy-related CO_2_ emissions globally. Given the overall carbon budget and the current CO2 emissions trends from building operations, the global temperature could possibly rise above 2 °C^47^. Geothermal energy provides significant opportunities to reduce the impacts of building operation, and it is receiving increasing attention in sustainable building design worldwide due to their reliability and other reported benefits. However, the lack of an integrated framework guiding the investigation of GSHP systems, along with the incapability of such well-known simulation tools as EnergyPlus in accurately capturing the ground thermal performance, hinders its application.

Guidelines are imperative for monitoring the performance of geothermal technologies, and the NREL guideline in the U.S. is the closest existing one to a comprehensive guide for GSHP applications. The NREL guideline categorises geothermal energy generation as part of *Facility Energy Production*, i.e., *Thermal Energy Production*. However, the monitoring protocol in the NREL guideline has yet to be sufficiently explained. For example, it does not address whether the thermal energy production of GSHP systems should be measured at the inlets or outlets of GSHPs, or what should be measured for the purpose of assessing the thermal energy production if the electricity consumption by GSHPs is not monitored separately. Therefore, amendments and improvements to the guideline are required for the energy monitoring of GSHP systems. Furthermore, to evaluate the actual performance of GSHP systems, an intelligent monitoring framework aligned with the improved guidelines needs to be developed.

While the use of GSHPs is becoming more widespread, precise performance simulation is also critical. These systems are typically designed using ground heat exchangers that are linked to heat pumps, and simulation can be used to predict the behaviour and the temperatures from the ground heat exchanger loops. However, most of the available simulation programs, such as TRNSYS (to simulate the transient systems’ behaviour) and EnergyPlus (whole-building energy modelling interface), are not sufficiently developed to accurately capture the ground thermal performance (*e.g.*, temperature distribution or heat capacity) or the interaction between GHEs. Accordingly, numerical design methods have been proposed to model and simulate the bore field heat exchanger response, among which ‘g-function’ developed by Eskilson is the most widely used method. This method was used by several studies and applications, including EnergyPlus-based applications such as GLHEPro and DesignBuilder. However, the g-function method is limited by several assumptions, including that the boreholes in the bore field are of equal dimension (i.e., length and radius), which may affect its accuracy. Cimmino and Bernier thus presented a method to compute g-functions for boreholes connected in any configuration of series and parallel connections. The research presented in this paper addresses GSHP utilisation through an integrated framework, including an overarching improved NREL guideline, a sensor-based monitoring prototype and instrument, and a g-function-based simulation approach.

## Conclusion

This research proposes amendments and improvements to the NREL guideline for monitoring geothermal energy by separating *Thermal Energy Net Production* from *Thermal Energy Gross Production*. *Thermal Energy Gross Production* is defined as the overall thermal energy generation that can be directly used for building operation, while *Thermal Energy Net Production* is defined as *Thermal Energy Gross Production* minus the *electrical consumption of geothermal heat pumps*. Without these improvements, the thermal energy generation could be overcounted by incorporating the energy conversion of electrical consumption of GSHPs. This research also proposes a monitoring prototype that uses thermal meters and temperature sensors, along with data loggers or a BMS for monitoring. With the key considerations of measurement metrics and installation locations determined based on the research objectives and in alignment with the improved monitoring guideline, thermal meters are installed at the outlet points of the supply side of the GSHP systems to monitor the thermal energy gross production. Additional temperature sensors are installed at the inlet points of the source side of the GSHP systems to monitor the water temperature for the purposes of evaluation and validation.

This paper also verifies the modified g-function method for simulating the performance of geothermal technologies. To predict thermal energy generation, this research uses an open-source application called Pygfunction, a Python-based package, for the computation of g-functions. This application overcomes the limitations and assumptions made by other popular computation methods. In particular, a Python script is created to calculate g-functions for any non-uniform, arbitrarily spaced bore field with mixed series and parallel interconnections between boreholes. A state-of-the-art case building located in Melbourne is used to demonstrate and validate the proposed framework. This building comprises 220 boreholes of non-uniform lengths connected in a mixed series–parallel configuration. The developed computational model is able to accommodate the series interconnections between boreholes and the non-uniformity of the boreholes in the field (as the boreholes are of varying lengths), and the computed g-functions are used to simulate geothermal production. The simulation results are then compared with the monitored data from the case study building. The findings reveal that: (1) the thermal energy generation during working days in July 2021 is close to the simulation results, with a difference of 2.2% in gross thermal energy production; (2) the average simulated temperature for July is 15.14 °C, and the average monitored temperature is 15.00 °C, resulting in a difference of 0.92% in inlet temperature. As such, the results and comparison between the simulated and monitored data demonstrate the efficacy of the proposed framework.

This research contributes to advancing GSHP utilisation by providing an integrated framework, including an overarching improved monitoring guideline, a monitoring prototype and instrument, and a g-function-based simulation approach. This research is subject to several limitations that should be considered when the methodology is employed in other studies. If the GSHP systems are designed for both heating and cooling, a longer study period spanning multiple seasons would be beneficial for evaluating how the system performs in both hot and cold times of the year. Based on long-term monitoring, more comprehensive statistical analysis can be conducted in future research. It will also be beneficial if monitoring guidelines provide guidance on minimum monitoring periods for different design scenarios and purposes, which can be addressed in future research. Another potential avenue of future research would be to investigate more and varied types of cases worldwide, as this would help draw more comprehensive conclusions based on the building type and renewable energy profile of the building under investigation.

## Supplementary Information

Supplementary Information.

## Data Availability

All data generated or analysed during this study are included in this published article and its supplementary information files. Please contact the corresponding author for data requests from this study.

## References

[1] United Nations Environment Programme (UNEP), Towards a zero-emission, efficient, and resilient buildings and construction sector: Global status report (accessed 6 May 2020); https://wedocs.unep.org/bitstream/handle/20.500.11822/34572/GSR_ES.pdf?sequence=3&isAllowed=y (2020).

[2] Keyßer LT, Lenzen M (2021). 1.5 C degrowth scenarios suggest the need for new mitigation pathways. Nat. Commun..

[3] Lund JW, Toth AN (2021). Direct utilization of geothermal energy 2020 worldwide review. Geothermics.

[4] Huang S (2012). Geothermal energy in China. Nat. Clim. Chang..

[5] United Nations Economic Commission for Europe (UNECE), High Performance Buildings and Climate (accessed 9 September 2022); https://unece.org/unece-and-sdgs/high-performance-buildings-and-climate (2022).

[6] Lu Q, Narsilio GA, Aditya GR, Johnston IW (2017). Economic analysis of vertical ground source heat pump systems in Melbourne. Energy.

[7] Geoscience Australia. Geothermal energy resources (accessed 29 September 2021); http://www.ga.gov.au/scientific-topics/energy/resources/geothermal-energy-resources (2018).

[8] Li HX, Edwards DJ, Hosseini MR, Costin GP (2020). A review on renewable energy transition in Australia: An updated depiction. J. Clean. Prod..

[9] Kurnia JC, Shatri MS, Putra ZA, Zaini J, Caesarendra W, Sasmito AP (2022). Geothermal energy extraction using abandoned oil and gas wells: Techno-economic and policy review. Int. J. Energy Res..

[10] Esen H, Inalli M, Esen M (2007). A techno-economic comparison of ground-coupled and air-coupled heat pump system for space cooling. Build. Environ..

[11] Kapıcıoğlu A, Esen H (2022). Economic and environmental assessment of ground source heat pump system: The case of Turkey. Sustain. Energy Technol. Assess..

[12] Self S, Reddy B, Rosen M (2013). Geothermal heat pump systems: Status review and comparison with other heating options. Appl. Energy.

[13] Ruiz-Calvo F, De Rosa M, Monzó P, Montagud C, Corberán JM (2016). Coupling short-term (B2G model) and long-term (g-function) models for ground source heat exchanger simulation in TRNSYS: Application in a real installation. Appl. Therm. Eng..

[14] Eswiasi A, Mukhopadhyaya P (2020). Critical review on efficiency of ground heat exchangers in heat pump systems. Clean Technol..

[15] Lee CK, Lam HN (2008). Computer simulation of borehole ground heat exchangers for geothermal heat pump systems. Renew. Energy.

[16] Liu Z, Liu Y, He BJ, Xu W, Jin G, Zhang X (2019). Application and suitability analysis of the key technologies in nearly zero energy buildings in China. Renew. Sustain. Energy Rev..

[17] Soni SK, Pandey M, Bartaria VN (2016). Hybrid ground coupled heat exchanger systems for space heating/cooling applications: A review. Renew. Sustain. Energy Rev..

[18] Qi Z, Gao Q, Liu Y, Yan YY, Spitler JD (2014). Status and development of hybrid energy systems from hybrid ground source heat pump in China and other countries. Renew. Sustain. Energy Rev..

[19] Yu, Y. & Olson, G. Ground Source Heat Pump Systems. Handbook of Energy Systems in Green Buildings, pp. 393–472 (2018).

[20] Miao, R., Yu, Y., Hu, X., & Sirotiak, T. Long-Term Monitoring and Simulation of a Vertical Closed-Loop Ground Source Heat Pump System Used in the Cold Climate of the U.S. The Free Library (July, 1) (2019). https://www.thefreelibrary.com/Long-TermMonitoringandSimulationofaVerticalClosed-LoopGround...-a0616448782

[21] Lu Y, Hooman K, Atrens AD, Russell H (2017). An experimental facility to validate ground source heat pump optimisation models for the Australian climate. Energies.

[22] Aditya, G. R., Narsilio, G. A., Johnston, I. W. & Disfani, M. M. (2018) September. Full-scale instrumented residential ground source heat pump systems in Melbourne, Australia. In *International Symposium on Energy Geotechnics* (pp. 185–191). Springer, Cham.

[23] Yebiyo, M., Maidment, G., Paurine, A. & Day, A. Metering measurement challenges & monitoring of a large scale ground source heat pump (GSHP) system. In *American Society of Heating Refrigeration and Air-conditioning Engineers (ASHRAE) winter Conference.*

[24] Spitler, J. D., Berglöf, K., Mazzotti-Pallard, W. & Witte, H. Guideline for Calculation of Uncertainties – Final Document. IEA HPT Annex 52 – Long-term performance monitoring of GSHP systems serving commercial, institutional and multi-family buildings. 10.23697/m2em-xq83 (2021).

[25] China Academy of Building Research, Evaluation Standard for Application of Renewable Energy in Buildings. In *GB/T 50801–2013*; China Architecture and Building Press: Beijing, China (2013).

[26] Tsagarakis KP, Efthymiou L, Michopoulos A, Mavragani A, Anđelković AS, Antolini F, Bacic M, Bajare D, Baralis M, Bogusz W, Burlon S (2020). A review of the legal framework in shallow geothermal energy in selected European countries: Need for guidelines. Renew. Energy.

[27] Barley, D., Deru, M., Pless, S. & Torcellini, P. Procedure for Measuring and Reporting Commercial Building Energy Performance, National Renewable Energy Laboratory (NREL) report (NREL/TP-550–38601). (2005).

[28] Hong T, Chou SK, Bong TY (2000). Building simulation: An overview of developments and information sources. Build. Environ..

[29] Arteconi A, Brandoni C, Rossi G, Polonara F (2013). Experimental evaluation and dynamic simulation of a ground coupled heat pump for a commercial building. Int. J. Energy Res..

[30] Noye S, Martinez RM, Carnieletto L, De Carli M, Aguirre AC (2022). A review of advanced ground source heat pump control: Artificial intelligence for autonomous and adaptive control. Renew. Sustain. Energy Rev..

[31] Luo J, Rohn J, Xiang W, Bertermann D, Blum P (2016). A review of ground investigations for ground source heat pump (GSHP) systems. Energy Build..

[32] Sarbu I, Sebrachievici C (2014). General review of ground-srouce heat pump systems for heating and cooling of buildings. Energy Build..

[33] EnergyPlus, EnergyPlus engineering reference. The reference to energyplus calculations, University of Illinois and the Ernest Orlando Lawrence Berkeley National Laboratory (2013).

[34] Cazorla-Marín A, Montagud-Montalvá C, Tinti F, Corberán JM (2020). A novel TRNSYS type of a coaxial borehole heat exchanger for both short and mid term simulations: B2G model. Appl. Therm. Eng..

[35] Cimmino M (2018). Fast calculation of the g-functions of geothermal borehole fields using similarities in the evaluation of the finite line source solution. J. Build. Perform. Simul..

[36] Eskilson P (1987). Thermal Analysis of Heat Extraction Boreholes.

[37] Cimmino M (2019). Semi-Analytical Method for g-Function Calculation of bore fields with series- and parallel-connected boreholes. Sci. Technol. Built Environ..

[38] ASHRAE, Heating, Ventilating, and Air-Conditioning Applications (SI Edition), American Society of Heating, Refrigerating and Air-Conditioning Engineers (2015).

[39] Yavuzturk C, Spitler JD (2000). Comparative study of operating and control strategies for hybrid ground-source heat pump systems using a short time step simulation model. ASHRAE Trans..

[40] Daniel, E. F. & Simon, J. R. Modeling Ground Source Heat Pump Systems in a Building Energy Simulation Program (EnergyPlus). In *IBPSA Building Simulation 2005.*

[41] DesignBuilder. *What is DesignBuilder?*, https://designbuilder.com.au/ (2021).

[42] Marcotte D, Pasquier P (2014). Unit-response function for ground heat exchanger with parallel, series or mixed borehole arrangement. Renew. Energy.

[43] Lamarche L, Beauchamp B (2007). A new contribution to the finite line-source model for geothermal boreholes. Energy Build..

[44] Cimmino M, Bernier M (2014). A semi-analytical method to generate g-functions for geothermal bore fields. Int. J. Heat Mass Transf..

[45] Cimmino, M. pygfunction: An open-source toolbox for the evaluation of thermal response factors for geothermal borehole fields. In *In Proceedings of eSim 2018, the 10*^*th*^* Conference of IBPSA.* pp. 492–501.

[46] Cimmino, M. pygfunction (accessed 30 November 2022); https://github.com/MassimoCimmino/pygfunction

[47] Climate Change Authority, A Global Emissions Budget For 2 Degrees or Less (accessed 9 September 2022); https://www.climatechangeauthority.gov.au/sites/default/files/2020-06/Target-Progress-Review/Targets%20and%20Progress%20Review%20Final%20Report_Chapter%203.pdf (2014).

